# Obinutuzumab: A Novel Anti-CD20 Monoclonal Antibody for Chronic Lymphocytic Leukemia

**DOI:** 10.6004/jadpro.2015.6.4.7

**Published:** 2015-07-01

**Authors:** Sarah S. Evans, Amber B. Clemmons

**Affiliations:** Georgia Regents Medical Center, Augusta, Georgia, and University of Georgia College of Pharmacy, Augusta, Georgia

The management of chronic lymphocytic leukemia (CLL), a malignant disorder of the lymphoid lineage (primarily B cells), has changed considerably in recent years. The development of agents targeting CD20, a transmembrane calcium channel protein expressed on B cells, was a significant milestone in the management of various lymphomas and leukemias (Cheson & Leonard, 2008; Byrd, Jones, Woyach, Johnson, & Flynn, 2014).

Rituximab (Rituxan), the first anti-CD20 monoclonal antibody, was originally approved by the US Food and Drug Administration (FDA) in 1997 for the treatment of non-Hodgkin lymphoma and subsequently for CLL. The German CLL8 study compared fludarabine and cyclophosphamide with or without rituximab in 761 previously untreated patients with CLL and demonstrated significant improvements in complete response, median progression-free survival (PFS), and overall survival (OS) in those who received rituximab (Hallek et al., 2010). This study thus established chemoimmunotherapy as the standard of care in young, symptomatic patients with CLL.

A second anti-CD20 monoclonal antibody, ofatumumab (Arzerra), was granted accelerated approval by the FDA in 2009 for patients with fludarabine-refractory/alemtuzumab-refractory CLL and subsequently in 2014 for previously untreated patients with CLL unable to tolerate fludarabine-based chemotherapy (Wierda et al., 2010, GlaxoSmithKline, 2014). This updated indication was based on a randomized trial comparing ofatumumab plus chlorambucil vs. chlorambucil alone in 447 treatment-naive patients with CLL (Hillmen et al., 2013). Overall response and PFS were significantly longer in the ofatumumab-chlorambucil group.

In November 2013, the third anti-CD20 monoclonal antibody, obinutuzumab (Gazyva), was approved by the FDA in combination with chlorambucil for patients with previously untreated CLL. The growing number of targeted immunotherapies in CLL, including obinutuzumab, may represent a paradigm shift in the management of this disease.

## DRUG CLASS AND MOLECULAR TARGET

CD20 is a transmembrane calcium channel involved in B-cell activation, proliferation, and differentiation (Cheson & Leonard, 2008). Type I anti-CD20 monoclonal antibodies, rituximab and ofatumumab, lead to complement-dependent cytotoxicity (CDC), stimulation of signaling leading to apoptosis, and antibody-dependent cell-mediated cytotoxicity (ADCC) through the recruitment of immune mediator cells (Herter et al., 2013).

Obinutuzumab is a type II, fully humanized anti-CD20 monoclonal antibody that binds in a unique conformation to the protein epitope on the CD20 channel, which partially overlaps with the section recognized by rituximab (Bologna et al., 2011). Due to its glycoengineered design, obinutuzumab has an increased affinity for natural killer cells, macrophages, and dendritic cells, which allows for greater ADCC than rituximab (Golay et al., 2013; Mössner et al., 2010). Although obinutuzumab does not stabilize CD20 in lipid rafts and therefore has less CDC, it has more effective direct B-cell apoptosis than rituximab via activation of polymorphonuclear neutrophils, which results in phagocytosis and cell death (Golay et al., 2013; Mössner et al., 2010). The broadened mechanism of action of obinutuzumab, in contrast to the mechanisms of rituximab and ofatumumab, is theorized to provide greater efficacy (Bologna et al., 2011; Golay et al., 2013).

## STUDY RESULTS

The FDA approval of obinutuzumab was based on a randomized, open-label, phase III study conducted in 781 adult patients with previously untreated CD20-positive CLL with a Cumulative Illness Rating Scale (CIRS) score of > 6, indicating a higher number of baseline comorbidities (Goede et al., 2014). Patients were randomized to receive one of three treatments: chlorambucil monotherapy, obinutuzumab with chlorambucil, or rituximab with chlorambucil.

Patients in the obinutuzumab/chlorambucil arm demonstrated significant PFS benefit compared with both chlorambucil alone and rituximab-chlorambucil (26.7 months vs. 11.1 months and 15.2 months, respectively; *p* < 0.001 for both comparisons). Obinutuzumab/chlorambucil had a significant OS benefit over chlorambucil monotherapy (death rates 9% vs. 20%, respectively; *p* = .002); however, there was no significant OS benefit with obinutuzumab/chlorambucil vs. rituximab/chlorambucil (death rates 8% vs. 12%, respectively; *p* = .08). A PFS benefit with obinutuzumab/chlorambucil was demonstrated regardless of age, gender, lymphocyte count, or CIRS score but was not demonstrated in patients with the poor prognostic cytogenetic marker del(17p).

## DOSING AND ADMINISTRATION

Prior to administration of obinutuzumab, patients should be evaluated for tumor lysis syndrome risk and hepatitis B reactivation. All patients should be tested for hepatitis B surface antigen (HBsAg) and hepatitis B core antibody (anti-HBc). If either serology is positive, consultation with an experienced physician in the management of hepatitis B is warranted to evaluate for appropriate prophylaxis and monitoring prior to initiating therapy with obinutuzumab.

The recommended dose of obinutuzumab is 1,000 mg (except in cycle 1, where 100 mg is administered on day 1 and 900 mg on day 2) as an intravenous (IV) infusion through a dedicated line (Table 1). The 100-mg dose is prepared in 100 mL of normal saline, whereas the 900-mg and 1,000-mg doses are prepared in 250 mL of normal saline (and are *never* administered as a push or bolus).

**Table 1:**
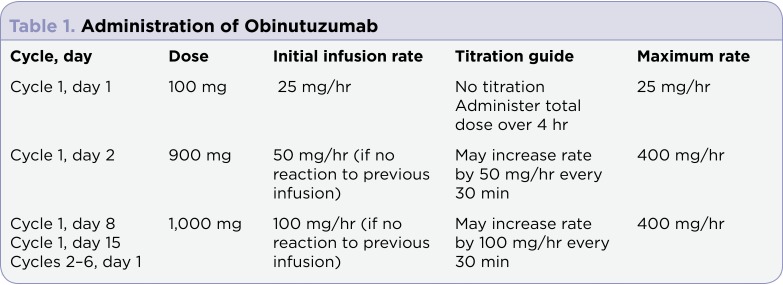
Administration of Obinutuzumab

In the first cycle, patients receive three 1,000-mg doses (with the first dose split over days 1 and 2 and the second and third doses given on days 8 and 15, respectively). Patients then receive 1,000 mg every 28 days for five additional cycles, for a total of six cycles. Obinutuzumab is approved in combination with chlorambucil, which is administered as 0.5 mg/kg orally on days 1 and 15 of each cycle (Genentech, 2014).

Premedication with acetaminophen (650–1,000 mg) is required before each dose. Prior to the doses on days 1 and 2 of cycle 1, all patients should receive an IV glucocorticoid (20 mg of dexamethasone or 80 mg of methylprednisolone) and an antihistamine such as diphenhydramine (50 mg). For patients who experience an infusion reaction during cycle 1 (day 1 or 2), subsequent infusions require premedication with an antihistamine. Also, if the reaction was grade 3 or the patient has a lymphocyte count above 25 × 10^9^/L, an IV glucocorticoid is also required before subsequent infusions (Genentech, 2014). Advanced practitioners should ensure that acetaminophen and antihistamine are administered 30 minutes prior and the glucocorticoid 1 hour prior to the start of the obinutuzumab infusion. Notably, hydrocortisone is not efficacious in reducing the incidence of infusion reactions and therefore is not recommended by the manufacturer (Genentech, 2014). Management of infusion-related reactions is detailed in Table 2.

**Table 2:**
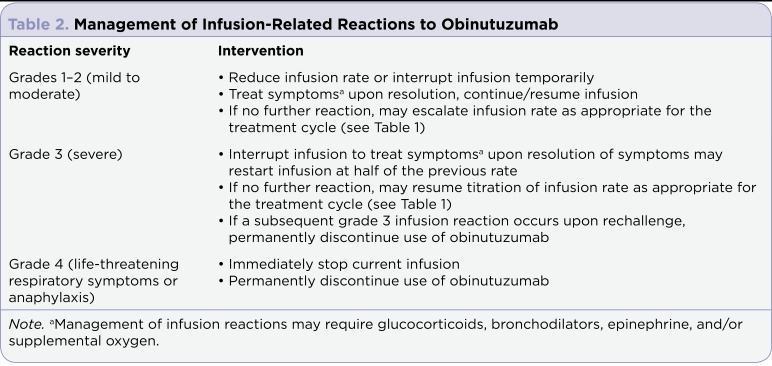
Management of Infusion-Related Reactions to Obinutuzumab

In addition to prophylaxis for infusion-related reactions, antibacterial prophylaxis is strongly recommended throughout the duration of treatment for patients with neutropenia; antiviral and antifungal prophylaxis should also be considered. Patients with a high tumor burden or an elevated lymphocyte count > 25 × 10^9^/L should receive prophylaxis for tumor lysis syndrome with allopurinol and hydration at least 12 hours prior to the start of the first dose of obinutuzumab (Genentech, 2014). Interruption in treatment should be considered for any patient with an infection, grade 3 or higher hematologic toxicity, or grade 2 or higher nonhematologic toxicity (Genentech, 2014).

Obinutuzumab has not been studied in patients with creatinine clearance < 30 mL/min, hepatic impairment, or younger than age 18. No formal drug interaction studies have been conducted; however, immunization with live vaccines during treatment and until recovery of B-cell lineage is not recommended (Genentech, 2014).

## ADVERSE EFFECTS

The following adverse reactions occurred in more than 10% of clinical trial participants (n = 240) who received obinutuzumab with chlorambucil: infusion reactions (69%; 21% grade 3/4), neutropenia (40%; 34% grade 3/4), thrombocytopenia (15%; 11% grade 3/4), anemia (12%), and pyrexia and cough (10% each). Laboratory abnormalities (> 20%) were also reported, including hypocalcemia, hyperkalemia, hyponatremia, increases in serum creatinine and liver function tests, and hypoalbuminemia (Goede et al., 2014).

Infusion reactions are the most common adverse effects reported with the use of obinutuzumab. Signs and symptoms can include hypotension; tachycardia; and respiratory symptoms such as dyspnea, wheezing, bronchospasm, irritation of the throat and larynx, and laryngeal edema.

After the first dose of obinutuzumab, the incidence of infusion reactions drops significantly (< 3%). Furthermore, in clinical trials, no grade 3 or 4 infusion reactions occurred after the first full dose. In patients who are not at risk for hypertensive crisis, advanced practitioners should consider withholding antihypertensive agents 12 hours prior to and during administration of obinutuzumab.

Due to the risk of thrombocytopenia and hemorrhage, consideration should be given to withholding concomitant medications that may increase the risk of bleeding, especially during the first cycle. Approximately 5% of patients experienced acute thrombocytopenia within 24 hours of infusion, and all fatal hemorrhagic events occurred during the first cycle.

Obinutuzumab carries two black box warnings: hepatitis B reactivation and progressive multifocal leukoencephalopathy (PML), which results from infection with the JC (John Cunningham) virus. Patients with new onset or alterations in preexisting neurologic symptoms should be evaluated promptly, and those diagnosed with PML should not receive further treatment with obinutuzumab.

## IMPLICATIONS FOR THE ADVANCED PRACTITIONER

Overall, anti-CD20 therapies have significantly improved outcomes for patients with CLL and form an integral component to the backbone of therapy for these patients. Based on the available clinical evidence, obinutuzumab is recommended as first-line treatment for elderly patients ≥ 70 years old or younger patients with multiple comorbid conditions who do not have del(11q) or del(17p) cytogenetic mutations (National Comprehensive Cancer Network, 2014).

If a patient is deemed suitable for obinutuzumab therapy, advanced practitioners should be cognizant of the risks of hepatitis B reactivation, PML, infusion reactions, and infections before initiating treatment. Thus, appropriate premedications prior to infusions and agents for antimicrobial prophylaxis should be utilized. It is also essential to obtain a baseline hepatitis B panel before initiating treatment with obinutuzumab, as reactivations associated with anti-CD20 monoclonal antibodies have resulted in hepatic failure and death (Hwang et al., 2015). Further research in CLL and other disease states will enhance our understanding of the safety and efficacy regarding obinutuzumab and allow advanced practitioners to optimize treatment for these patients.

## References

[1] Bologna Luca, Gotti Elisa, Manganini Massimiliano, Rambaldi Alessandro, Intermesoli Tamara, Introna Martino, Golay Josée (2011). Mechanism of action of type II, glycoengineered, anti-CD20 monoclonal antibody GA101 in B-chronic lymphocytic leukemia whole blood assays in comparison with rituximab and alemtuzumab.. *Journal of immunology (Baltimore, Md. : 1950)*.

[2] Byrd John C., Jones Jeffrey J., Woyach Jennifer A., Johnson Amy J., Flynn Joseph M. (2014). Entering the era of targeted therapy for chronic lymphocytic leukemia: impact on the practicing clinician.. *Journal of clinical oncology : official journal of the American Society of Clinical Oncology*.

[3] Cheson Bruce D., Leonard John P. (2008). Monoclonal antibody therapy for B-cell non-Hodgkin's lymphoma.. *The New England journal of medicine*.

[4] (2014). Obinutuzumab (Gazyva) package insert.. Genentech, Inc..

[5] (2014). Ofatumumab (Arzerra) package insert.. GlaxoSmithKline..

[6] Goede Valentin, Fischer Kirsten, Busch Raymonde, Engelke Anja, Eichhorst Barbara, Wendtner Clemens M., Chagorova Tatiana, de la Serna Javier, Dilhuydy Marie-Sarah, Illmer Thomas, Opat Stephen, Owen Carolyn J., Samoylova Olga, Kreuzer Karl-Anton, Stilgenbauer Stephan, Döhner Hartmut, Langerak Anton W., Ritgen Matthias, Kneba Michael, Asikanius Elina, Humphrey Kathryn, Wenger Michael, Hallek Michael (2014). Obinutuzumab plus chlorambucil in patients with CLL and coexisting conditions.. *The New England journal of medicine*.

[7] Golay Josée, Da Roit Fabio, Bologna Luca, Ferrara Claudia, Leusen Jeanette H., Rambaldi Alessandro, Klein Christian, Introna Martino (2013). Glycoengineered CD20 antibody obinutuzumab activates neutrophils and mediates phagocytosis through CD16B more efficiently than rituximab.. *Blood*.

[8] Hallek M., Fischer K., Fingerle-Rowson G., Fink A. M., Busch R., Mayer J., Hensel M., Hopfinger G., Hess G., von Grünhagen U., Bergmann M., Catalano J., Zinzani P. L., Caligaris-Cappio F., Seymour J. F., Berrebi A., Jäger U., Cazin B., Trneny M., Westermann A., Wendtner C. M., Eichhorst B. F., Staib P., Bühler A., Winkler D., Zenz T., Böttcher S., Ritgen M., Mendila M., Kneba M., Döhner H., Stilgenbauer S. (2010). Addition of rituximab to fludarabine and cyclophosphamide in patients with chronic lymphocytic leukaemia: a randomised, open-label, phase 3 trial.. *Lancet (London, England)*.

[9] Herter Sylvia, Herting Frank, Mundigl Olaf, Waldhauer Inja, Weinzierl Tina, Fauti Tanja, Muth Gunter, Ziegler-Landesberger Doris, Van Puijenbroek Erwin, Lang Sabine, Duong Minh Ngoc, Reslan Lina, Gerdes Christian A., Friess Thomas, Baer Ute, Burtscher Helmut, Weidner Michael, Dumontet Charles, Umana Pablo, Niederfellner Gerhard, Bacac Marina, Klein Christian (2013). Preclinical activity of the type II CD20 antibody GA101 (obinutuzumab) compared with rituximab and ofatumumab in vitro and in xenograft models.. *Molecular cancer therapeutics*.

[10] Hillmen P., Robak T., Janssens A., Govindbabu K., Grosicki S., Mayer J., Offner F. (2013). Ofatumumab + chlorambucil versus chlorambucil alone in patients with untreated chronic lymphocytic leukemia (CLL): Results of the phase III study Complement 1 (OMB110911) [Abstract 528].. * Blood (ASH Annual Meeting Abstracts)*.

[11] Hwang Jessica P., Somerfield Mark R., Alston-Johnson Devena E., Cryer Donna R., Feld Jordan J., Kramer Barnett S., Sabichi Anita L., Wong Sandra L., Artz Andrew S. (2015). Hepatitis B Virus Screening for Patients With Cancer Before Therapy: American Society of Clinical Oncology Provisional Clinical Opinion Update.. *Journal of clinical oncology : official journal of the American Society of Clinical Oncology*.

[12] Mössner Ekkehard, Brünker Peter, Moser Samuel, Püntener Ursula, Schmidt Carla, Herter Sylvia, Grau Roger, Gerdes Christian, Nopora Adam, van Puijenbroek Erwin, Ferrara Claudia, Sondermann Peter, Jäger Christiane, Strein Pamela, Fertig Georg, Friess Thomas, Schüll Christine, Bauer Sabine, Dal Porto Joseph, Del Nagro Christopher, Dabbagh Karim, Dyer Martin J. S., Poppema Sibrand, Klein Christian, Umaña Pablo (2010). Increasing the efficacy of CD20 antibody therapy through the engineering of a new type II anti-CD20 antibody with enhanced direct and immune effector cell-mediated B-cell cytotoxicity.. *Blood*.

[13] (2014). Non-Hodgkin’s Lymphoma. v4.2014.. National Comprehensive Cancer Network..

[14] Wierda William G., Kipps Thomas J., Mayer Jirí, Stilgenbauer Stephan, Williams Cathy D., Hellmann Andrzej, Robak Tadeusz, Furman Richard R., Hillmen Peter, Trneny Marek, Dyer Martin J. S., Padmanabhan Swami, Piotrowska Magdalena, Kozak Tomas, Chan Geoffrey, Davis Randy, Losic Nedjad, Wilms Joris, Russell Charlotte A., Osterborg Anders (2010). Ofatumumab as single-agent CD20 immunotherapy in fludarabine-refractory chronic lymphocytic leukemia.. *Journal of clinical oncology : official journal of the American Society of Clinical Oncology*.

